# Generation of Recombination Activating Gene-1-Deficient Neonatal Piglets: A Model of T and B Cell Deficient Severe Combined Immune Deficiency

**DOI:** 10.1371/journal.pone.0113833

**Published:** 2014-12-01

**Authors:** Tetsuya Ito, Yutaka Sendai, Satoshi Yamazaki, Marie Seki-Soma, Kensuke Hirose, Motoo Watanabe, Kazuo Fukawa, Hiromitsu Nakauchi

**Affiliations:** 1 Pig Breeding Laboratory, Central Research Institute for Feed and Livestock, ZEN-NOH (National Federation of Agricultural Co-operative Associations), Hokkaido, Japan; 2 Biological Sciences Section, Central Research Institute for Feed and Livestock, ZEN-NOH (National Federation of Agricultural Co-operative Associations), Ibaraki, Japan; 3 Division of Stem Cell Therapy, The Institute of Medical Science, The University of Tokyo, Tokyo, Japan; 4 Zen-noh Institute of Animal Health, ZEN-NOH (National Federation of Agricultural Co-operative Associations), Chiba, Japan; 5 Japan Science and Technology Agency, Exploratory Research for Advanced Technology (ERATO), Nakauchi Stem Cell and Organ Regeneration Project, Tokyo, Japan; Institut de Recherches Cliniques de Montréal (IRCM), Canada

## Abstract

Although severe combined immune deficiency (SCID) is a very important research model for mice and SCID mice are widely used, there are only few reports describing the SCID pig models. Therefore, additional research in this area is needed. In this study, we describe the generation of *Recombination activating gene-1* (*Rag-1*)-deficient neonatal piglets in Duroc breed using somatic cell nuclear transfer (SCNT) with gene targeting and analysis using fluorescence-activated cell sorting (FACS) and histology. We constructed porcine *Rag-1* gene targeting vectors for the Exon 2 region and obtained heterozygous/homozygous *Rag-1* knockout cell colonies using SCNT. We generated two *Rag-1*-deficient neonatal piglets and compared them with wild-type neonatal piglets. FACS analysis showed that *Rag-1* disruption causes a lack of Immunoglobulin M-positive B cells and CD3-positive T cells in peripheral blood mononuclear cells. Consistent with FACS analysis, histological analysis revealed structural defects and an absence of mature lymphocytes in the spleen, mesenteric lymph node (MLNs), and thymus in *Rag-1*-deficient piglets. These results confirm that *Rag-1* is necessary for the generation of lymphocytes in pigs, and *Rag-1*-deficient piglets exhibit a T and B cell deficient SCID (T-B-SCID) phenotype similar to that of rodents and humans. The T-B-SCID pigs with *Rag-1* deficiency generated in this study could be a suitably versatile model for laboratory, translational, and biomedical research, including the development of a humanized model and assessment of pluripotent stem cells.

## Introduction

The V(D)J recombination generates antibody and T cell receptor diversity [1], [2]. *Recombination activating gene-1* and *-2* (*Rag-1*, *-2*) were identified as activators of V(D)J recombination in NIH 3T3 fibroblasts grown on an artificial substrate [3]. Cotransfection of *Rag-1* and *Rag-2* into fibroblasts synergistically activates V(D)J recombination [4], and both Rag-1 and Rag-2 proteins are sufficient to perform V(D)J recombination, which requires DNA nicking (double-strand breaks) and hairpin formation at an early stage [5]. Moreover, it is established that *Rag-1* and *Rag-2* play a crucial role in lymphoid cell development. Both *Rag-1*- and *Rag-2*-deficient mice lack mature B and T lymphocytes because of the blockage of lymphocyte differentiation early in development [6], [7]. In humans, *Rag* mutations cause severe combined immunodeficiency (SCID) with a complete absence of both T and B cells (T-B-SCID) by a complete block of T and B cell differentiation and Omenn syndrome and granulomas by impaired V(D)J recombination [8]–[12].

Currently, SCID model mice, including *Rag*-deficient mice, are widely used in research. Even more severely immunodeficient models based on SCID mice, such as the nonobese diabetic/shi-scid (NOD/SCID), NOG (NOD/shi-scid/γ_c_null), NSG (NOD/scid/γ_c_^−/−^), BALB/c Rag-2^−/−^γ_c_^−/−^, and NOD/SCID/huBLT strains, are also used as a source for the xenotransplantation of human tissues and cells [13]–[17]. Immunodeficient mice lacking T, B, natural killer (NK) cells, and CD122^+^ plasmacytoid dendritic cells permit and maintain human cell grafts, such as hematopoietic stem cells and hepatocytes [18]–[20]. Therefore, humanized mice, with functional human hematopoietic, immune systems, and liver can be developed by transplantation of these mice with human hematopoietic stem cells or human hepatocytes [14]–[17], [21]–[23]. Advanced research on human-specific viruses, such as human immunodeficiency virus, Epstein-Barr virus, dengue fever, and hepatitis C virus, have been conducted using these humanized mice [23]–[25].

Because of their anatomic, nutritional, physiologic, and genetic similarities to humans, pigs are essential for biomedical research [26], [27]. The cloning of pigs by somatic cell nuclear transfer (SCNT) was established in 2000 [28]–[30]. Subsequently, SCNT combined with gene targeting has enabled the production of genetically modified pigs for use in xenotransplantation as human disease models and in regenerative medicine [31]–[34]. Some genetically modified pig models show greater similarities to human diseases than rodent models. For example, pig models of cystic fibrosis, retinitis pigmentosa, Type 2 diabetes, X-linked SCID, and familial adenomatous polyposis models have been established [35]–[39]. Regarding models of SCID, a T-B+NK- pig model of X-linked SCID was recently generated by targeted disruption of the *interleukin 2 receptor gamma chain (Il2rg) gene* [38], [40]. Moreover, the T-B-NK+ SCID model pigs that had *Rag-1/-2* inactivated using gene editing technology have been recently reported in 2014 [41], [42]. Although studies about SCID model pigs are beginning to be reported, there are only four reports to date. Therefore, additional research is necessary to establish pig SCID models. Our objective was to generate *Rag-1*-deficient neonatal piglets of Duroc breed using SCNT with gene targeting, and analyze their peripheral blood mononuclear cells (PBMCs) and histology.

## Materials and Methods

### Ethics statement

All animal experiments were approved by the Animal Care Committee of the ZEN-NOH Central Research Institute for Feed and Livestock, Tsukuba, Japan (Permit Number: 20091224). All studies conducted *in vivo* were performed using sodium pentobarbital or midazolam/medetomidine with a combination of isoflurane and nitrous oxide anesthesia, and all efforts were made to minimize suffering.

### Vector construction

Targeting vector construction was performed as previously described [43]. The porcine *Rag-1* gene targeting vector was designed using *Rag-1* Exon 2 (Accession Number: AB091392, GenBank: AB091392.1) in the National Center for Biotechnology Information database. The 3′ short homologous arm for the *Rag-1* KO vector was generated from a 1.5 kilo base pair (kb) fragment by polymerase chain reaction (PCR) using the forward primer 5′-GCGATGTGAAGTCAGTGTGC-3′ and the reverse primer 5′-CCTCATATCTGTACTTGAACTTGG-3′ and the pig genomic DNA of Duroc fetal fibroblasts. Likewise, the 5′ long homologous arm was generated from a 6.5 kb *Rag-1* gene fragment containing Exons 1 and 2 by PCR using the forward primer 5′-GCAGATGCAACTCCAATTCC-3′ and the reverse primer 5′-CTCAGACGGTGTTTCTGAGC-3′. The *Rag-1* heterozygous KO vector was constructed by the insertion of two homologous arms into the PGK-Neo/MC1-TK plasmid vector as previously described. The *Rag-1* homozygous KO PGK-Neo vector was modified to contain the antibiotic resistance gene CAG-blasticidin resistance gene (bsr).

### Preparation of *Rag-1* KO cells

The preparation of *Rag-1* KO cells was performed as previously described [43]. Original porcine fetal fibroblasts (PFF: T6-12) were isolated from a wild-type Duroc male fetus and cultured in minimum essential medium-α containing 10% fetal calf serum (10% FCS-MEMα; Invitrogen, Carlsbad, CA, USA) at 38.5°C in 5% CO_2_. Transfection of the *Rag-1* heterozygous KO vectors was performed by electroporation. PFFs (1×10^7^) were transfected with 5 µg of the *Rag-1* heterozygous KO vector at 220 V and 950 µF using a Gene Pulser apparatus (Bio-Rad Laboratories, Hercules, CA, USA). Transfected cells were cultured in 10% FCS-MEMα in a 6-well plate for 48 h. After incubation, the transfected cells were suspended with 400 µg/ml Geneticin (Invitrogen, Carlsbad, CA, USA) and 20 µM gancyclovir (Nacalai Tesque, Inc., Kyoto, Japan) for positive–negative selection. For the preparation of the *Rag-1* homozygous KO cells, *Rag-1* heterozygous KO porcine fetal fibroblasts (*Rag* ^+/−^ PFF) were isolated from a *Rag-1* heterozygous Duroc male fetus produced by nuclear transfer. These cells were then transfected with the *Rag-1* homozygous KO vector using the method described above. For the selection of homozygous KO cells, 10 µg/ml blasticidin (Invitrogen, Carlsbad, CA, USA) was added to the heterozygous KO selection method. After being cultured for 10 days, each cell colony was separated into two parts and then continuously cultured. After from 24 h to 48 h culture, one of the two-division cells was isolated and used for PCR analysis [43]. Positive KO cells were grown to confluence in a 75 cm^2^ flask and cryopreserved until SCNT.

### Oocyte collection and SCNT

All pigs of this study were raised in a specific pathogen free (SPF) environment. *In vivo* matured oocytes were collected from estrus-synchronized and superovulated gilts and sows treated with equine chorionic gonadotropin and human chorionic gonadotropin (Novartis Animal Health Inc., Tokyo, Japan) with/without prostaglandin F_2α_ (Asuka Pharmaceutical Co., Ltd., Tokyo, Japan) as described previously [44]–[46]. Oocytes were removed from cumulus cells in 1 mg/ml hyaluronidase in phosphate-buffered saline (PBS; Takara Bio Inc., Shiga, Japan) supplemented with 0.1% bovine serum albumin (Sigma-Aldrich, St.Louis, MO, USA). Enucleation and SCNT were performed using a procedure based on blind methods using a piezo-actuated system (PRIME TECH LTD., Ibaraki, Japan) previously described [28]. Oocytes injected with donor cells were electrically activated by a single direct current pulse at 1.5 kV/cm for 100 µs. Reconstructed embryos were transferred into porcine zygote medium-3 (PZM-3) supplemented with 5 µg/ml cytochalasin B (Sigma-Aldrich, St.Louis, MO, USA), and after a 2 h incubation at 38.5°C in 5% CO_2_, 5% O_2_, and 90% N_2_, the embryos were cultured in PZM-3 until embryo transfer [47]. Reconstructed embryos with clear cytoplasts were selected and surgically transferred into the oviducts of estrus-synchronized recipient gilts. All the *Rag-1*-deficient neonatal piglets produced during this research were euthanized, along with age-matched two wild-type piglets, for analysis.

### DNA extraction and genomic PCR

The original porcine fetal fibroblasts (T6-12), *Rag-1* heterozygous KO cells (#95-2, *Rag* ^+/−^ PFF), and *Rag-1* homozygous KO cells (#3-55, *Rag* ^−/−^ PFF), as well as the tail tissues of the *Rag-1*-deficient piglets (*Rag* ^−/−^ No. 1 and No. 2) and wild-type piglets (WT No. 1 and No. 2), were used for genomic PCR analysis. Genomic DNA extraction and PCRs were conducted using a QuickGene DNA Tissue Kit (Fujifilm Corp., Tokyo, Japan) and QuickTaq (Toyobo Co., Ltd., Osaka, Japan) according to the manufacturers' protocols. The primers used to detect the targeted allele 1 vector and heterozygous KO were: P1 (5′-TAGTACTTGGACTGCCTGGC-3′) and P2 (5′-GGCATGCATCGATAGATCTCG-3′). The PCR conditions were: 95°C for 1 min, 57°C for 1 min, and 72°C for 2 min for 35 cycles. The primers used to detect the targeted allele 2 vector and homozygous KO were: P1 and P3 (5′-GGTCCCTCGAAGAGGTTCACTAG-3′). The primers used to confirm *Rag-1* deficiency were: Pc1 (5′-TTCGCCGACAAAGAAGAAGG-3′) and Pc2 (5′-CTTGCAGCATAGTTCAGAGTTAGG-3′). The PCR conditions were the same. After PCR, 7 µl of each PCR products was loaded on to a 1% agarose gel and separated, and the ethidium bromide-stained gel was photographed.

### FACS

PBMCs were analyzed by FACS as previously described [48]. Peripheral whole blood was collected from each wild-type and *Rag-1*-deficient newborn piglet and immediately transferred to a collection tube supplemented with ethylenediaminetetraacetic acid to prevent coagulation. Gradient centrifugation was used to separate PBMCs. After PBMCs were washed with PBS, they were stained for 30 min at 4°C using a phycoerythrin-conjugated mouse anti-porcine CD3ε antibody (Beckman Coulter, Inc., Brea, CA, USA) and a fluorescein isothiocyanate-conjugated mouse anti-human CD19 antibody (Affimetrix, Inc., Santa Clara, CA, USA). Samples were analyzed on a flow cytometer (Becton, Dickinson and Company, Flanklin Lakes, NJ, USA).

### Histological analysis

Spleen, MLNs, and thymus samples were collected from both wild-type and *Rag-1*-deficient neonatal piglets. These tissues were fixed in 4% paraformaldehyde (Wako Pure Chemical Industries, Ltd., Osaka, Japan) and embedded in paraffin blocks. Thereafter, tissue samples were cut at 4 µm and stained with hematoxylin and eosin (H&E). Histologic sections were evaluated under a microscope.

## Results

### Preparation of *Rag-1* KO fetus fibroblast cells

**Figure 1:**
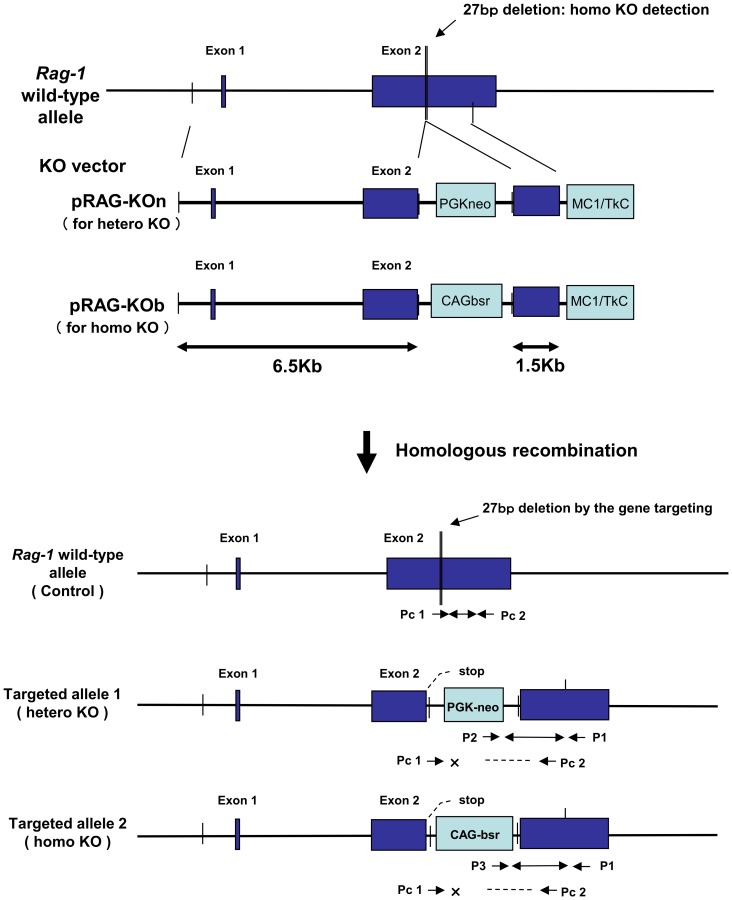
The porcine *Recombination activating gene-1* gene targeting scheme and primer designs. The porcine *Recombination activating gene-1* (*Rag-1*) gene, *Rag-1* gene targeting vector structure, and targeted *Rag-1* knockout (KO) allele are shown. The antibiotic resistance gene CAG-blasticidin resistance gene (bsr) were inserted into Exon 2 of the *Rag-1* gene with a 27-base pair (bp) deletion of part of Exon 2. The polymerase chain reaction (PCR) primers (P1 and P3) used to identify the targeted cell clones and *Rag-1* KO fetal fibroblasts and detect KO clone piglets are indicated by arrows. The PCR primers (Pc1 and Pc2) were used to identify homozygous KO piglets and detect the 27-bp deletion region caused by homologous recombination.

The *Rag-1* targeting vectors constructed and the gene targeting scheme are shown in Figure 1. The vector construct was designed to produce a 27-base pair (bp) deletion in Exon 2 including open reading frame by homologous recombination as a result of the antibiotic resistance gene cassette. Therefore, translation is stopped approximately 1.3 kb downstream from the initiator codon, and truncated *Rag-1* proteins are produced. Transfected cells possessing a DNA fragment from the inserted vector region of the antibiotic resistance gene to the 3′ short homologous arm region were amplified by PCR, whereas nontransfected cells were not. Nine of the 384 cell colonies obtained were PCR-positive for *Rag-1* heterozygous KO transfection. Among them, two colonies, #51-2 and #95-2, were successfully expanded and used for SCNT. Regarding *Rag-1* homozygous KO transfection, of the 288 colonies, 12 PCR-positive cell colonies were obtained, six of which were used for SCNT.

### Cloning and production of *Rag-1* KO neonatal piglets

**Figure 2:**
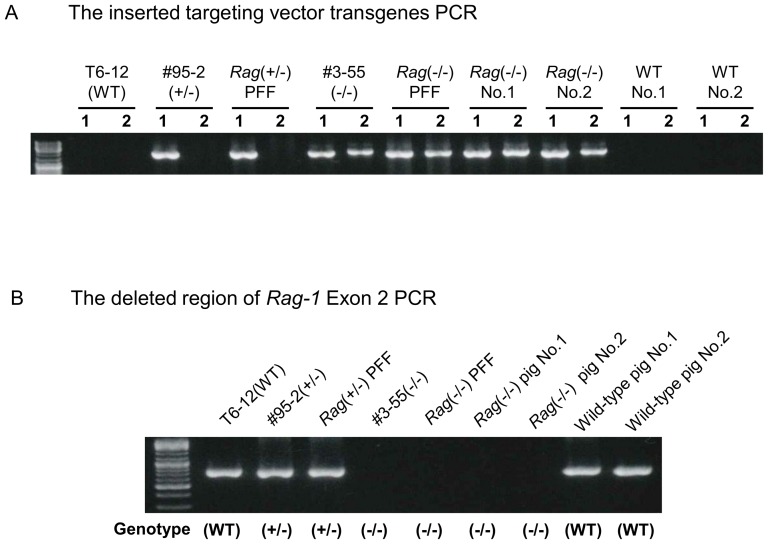
Polymerase chain reaction (PCR) analysis of the genomic DNA of *Recombination activating gene-1*-knockout fetal fibroblasts and tail tissues. (A) Polymerase chain reaction (PCR) analysis of *Recombination activating gene-1* (*Rag-1*)-knockout (KO) genomic DNA to detect the inserted vectors. The lane marked T6-12 displays results from the original porcine fetal fibroblasts used for preparation of the KO cells. The lanes marked WT displays results from the non-transgenic wild-type controls. Lanes marked #95-2 and *Rag* ^+/−^ PFF display results from *Rag-1* heterozygous KO cells and *Rag-1* heterozygous KO porcine fetal fibroblasts. Lanes marked #3-55 and *Rag* ^−/−^ PFF display results from *Rag-1* homozygous KO cells and *Rag-1* homozygous KO porcine fetal fibroblasts. Lanes marked *Rag* ^−/−^ No. 1 and No. 2 display the results from the tails of the two *Rag-1* homozygous KO neonatal piglets. Lanes marked WT No. 1 and No. 2 display results from the tails of non-transgenic wild-type control piglets. Primers P 1, P 2, and P 3 were used for PCRs. Lane 1 detects the heterozygous KO of targeted allele 1, and lane 2 detects the homozygous KO of targeted allele 2. (B) PCR of genomic DNA to demonstrate the deletion of the *Rag-1* region of Exon 2 by homologous recombination. WT is shown as a nontransgenic wild-type control. *Rag-1* heterozygous and homozygous KO are shown (^+/−^) and (^−/−^). The lane marked T6-12 displays results from the original porcine fetal fibroblasts used to prepare the KO cells. Lanes marked #95-2 and *Rag* ^+/−^ PFF display results from the *Rag-1* heterozygous KO cells and *Rag-1* heterozygous KO porcine fetal fibroblasts. Lanes marked #3-55 and *Rag* ^−/−^ PFF display results from the *Rag-1* homozygous KO cells and *Rag-1* homozygous KO porcine fetal fibroblasts. Lanes marked *Rag* ^−/−^ pig No. 1 and No. 2 display results from the tails of the *Rag-1* homozygous KO neonatal piglets. Lanes marked wild-type pig No. 1 and No. 2 display results from the tails of non-transgenic wild-type control piglets. The primer set consisting of Pc 1 and Pc 2 was used for the PCR analysis. The 27-base pair (bp) deletion detected in Exon 2 including open reading frame was caused by homologous recombination.

*Rag-1* heterozygous KO donor cells (#95-2) obtained from the original PFF (T6-12) by gene targeting were used for SCNT (Table 1: Step 1). The reconstructed embryos were cultured in PZM-3 medium for 2 days. Day 2 reconstructed embryos were transferred to one recipient gilt, and the pregnant gilt was used to obtain a *Rag-1* heterozygous KO fetus for the generation of *Rag-1* homozygous KO cells at a gestation of 33 days (Fig. 2: *Rag* ^+/−^ PFF). Next *Rag-1* homozygous KO donor cells (#3-55), derived from *Rag-1* heterozygous KO PFF by homologous recombination, were used to obtain *Rag-1* homozygous KO fetal fibroblast cells (Table 1: Step 2). The *Rag-1* homozygous KO fetal fibroblast cells generated were used to produce *Rag-1*-deficient newborn piglets (Fig. 2: *Rag* ^−/−^ PFF). A total of 526 reconstructed Day 1 and 2 embryos were mixed and transferred into four recipient gilts (Table 1: Step 3). Pregnancy was confirmed in three recipients using an ultrasound scanner at 26–28 days after embryo transfer. Eventually, two pregnant sows delivered two piglets by cesarean section.

**Table 1:** Production of *Recombination activating gene-1*-knockout pig fetuses or piglets by somatic cell nuclear transfer.

Step	Objective	Donor cells line	Transferred embryos	Recipient breed	Recipient No.	Pregnancy	KO Fetus or Piglet No.	KO Fetus or Piglet No./Transferred embryo (%)
1	*Rag-1* ^+/−^ PFF preparation	#95-2 (^+/−^)	20	Duroc	1	○	2	10.00
2	*Rag-1* ^−/−^ PFF preparation	#3-55(^−/−^)	49	Duroc	1	○	1	2.04
3	*Rag-1* ^−/−^ piglets generation	*Rag* ^−/−^ PFF	139	Duroc	1	○	1*	0.72
		*Rag* ^−/−^ PFF	130	Large White	1	×	-	-
		*Rag* ^−/−^ PFF	110	Duroc	1	○	1	0.91
		*Rag* ^−/−^ PFF	147	Large White	1	○	1	0.68

*: This fetus was aborted 88 days after embryo transfer.

Samples of genomic DNA were extracted from the original PFF (T6-12), heterozygous KO cells (#95-2, *Rag* ^+/−^ PFF), and homozygous KO cells (#3-55, *Rag* ^−/−^ PFF). Moreover, the tail tissues were obtained from the *Rag-1*-deficient (*Rag* ^−/−^ No. 1 and No. 2) and newborn wild-type piglets (WT No. 1 and No. 2) for genomic DNA extraction. Genomic PCR was employed to confirm *Rag-1*-deficiency and the presence of targeting vector transgenes (Fig. 2A). The targeting vector transgene was detected in *Rag-1*-deficient newborn piglets, which was in agreement with *Rag* ^−/−^ PFF donor cells, but was not detected in wild-type piglets. Moreover, the *Rag-1* Exon 2 region containing a 27 bp region deleted as a result of homologous recombination was present in wild-type piglets (Fig. 2B). On the other hand, *Rag-1*-deficient piglets did not possess the deleted 27 bp region of *Rag-1* Exon 2. The genomic PCRs of *Rag-1*-deficient piglets perfectly matched that of donor cells (#3-55, *Rag* ^−/−^ PFF). These results imply that the piglets generated by SCNT were cloned from *Rag-1*-deficient donor cells and possessed an inactivated *Rag-1* gene.

**Figure 3:**
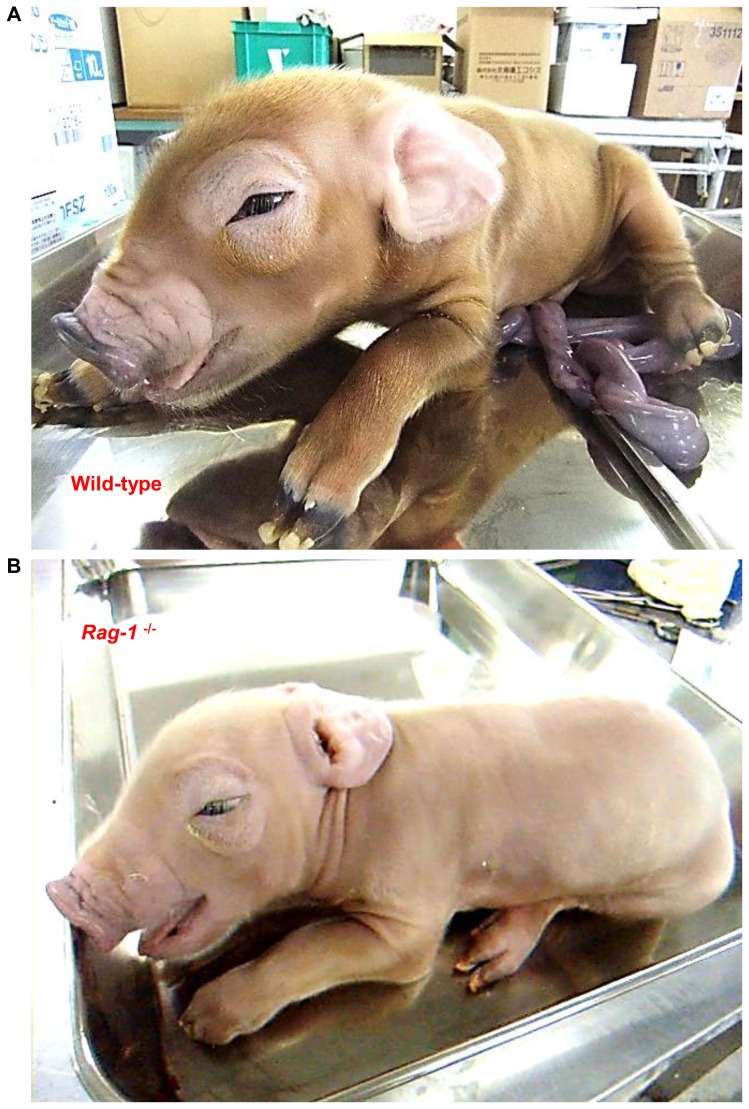
Photograph of *Recombination activating gene-1*-deficient neonatal piglets. (A) Photograph of wild-type (*Rag-1* ^+/+^) piglets. (B) Photograph of *Recombination activating gene-1* (*Rag-1*)^−/−^ piglets. Each was obtained by cesarean section 1 day before their expected date of birth.

The phenotype of *Rag-1*-deficient piglets is shown in Figure 3. Unlike wild-type Duroc piglets, which have a dark brown coat (Fig. 3A), *Rag-1*-deficient piglets exhibited hypopigmentation (Fig. 3B).

### FACS analysis of PBMC

**Figure 4:**
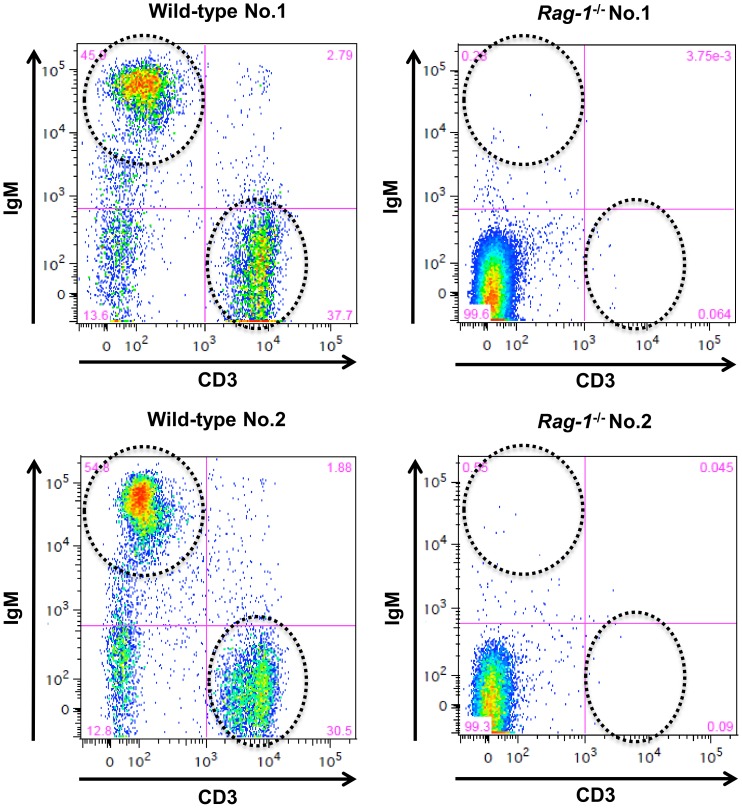
Fluorescence-activated cell sorting analysis of peripheral blood mononuclear cells. CD3 and CD19 mark differentiation of the T and B cell populations, respectively.

We used FACS to analyze differentiated lymphocyte populations among PBMCs using an anti-porcine CD3 antibody for mature T cells and an anti-human CD19 antibody for Immunoglobulin M (IgM)-positive B cells. The anti-human CD19 antibody used in FACS analysis reacted with porcine B cells. Wild-type (*Rag-1* ^+/+^) piglets possessed more than 30% CD3-positive T cells and 45% IgM-positive B cells among their PBMCs (Fig. 4). In contrast, CD3-positive T cells and IgM-positive B cells were not present (<1%) in *Rag-1* ^−/−^ piglets. Therefore, *Rag-1* deficiency prevents the differentiation of lymphocytes and causes a lack of IgM-positive B and mature T lymphocytes in pigs.

### Histological analysis of *Rag* ^−/−^ neonatal piglets

**Figure 5:**
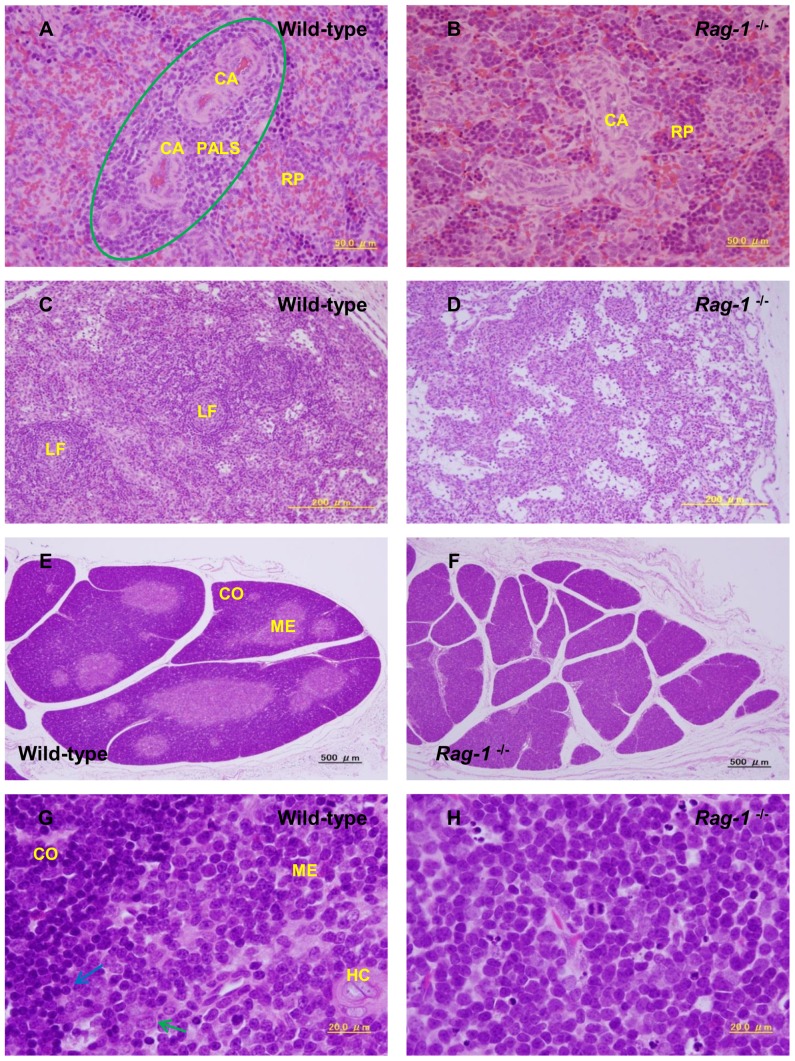
Histological analysis of *Recombination activating gene-1*-deficient piglets. (A and B) Histological analysis of the spleen (×400). The ellipse shows the white pulp lesion. Abbreviations: CA, central artery; PALS, periarterial lymphatic sheaths; RP, red pulp. (C and D) Histological analysis of the mesenteric lymph nodes (×100). Abbreviations: LF, lymphoid follicle. (E and F) Histological analysis of the thymus of *Recombination activating gene-1* (*Rag-1*)^−/−^ and wild-type piglets (×40). Abbreviations: CO, cortex; ME, medulla. (G and H) Histological analysis of the thymus (×1000). Panel G shows the limited medulla (ME) and cortex (CO). The blue and green arrows mark thymocytes and reticular cells, respectively. Abbreviation: HC, Hasseall's corpuscle.

Spleens, MLNs, and thymuses were histologically investigated by H&E staining (Fig. 5). In wild-type neonatal piglets, spleens clearly possessed white pulp, clusters of lymphocytes had formed around the central artery (Fig. 5A), and numerous lymphoid follicles were evident in MLNs (Fig. 5C). In contrast, the white pulp of the spleen and lymphoid follicles of MLNs were unclear and virtually devoid of small lymphocytes in *Rag-1* ^−/−^ neonatal piglets (Fig. 5B, D). Likewise, in wild-type piglets, the lobules of the thymus consisted of cortex and medulla and many lymphocytes were extant in the cortex region (Fig. 5E, G). On the other hand, there was no division between the cortex and medulla in the lobules of the thymus in *Rag-1* ^−/−^ piglets, and polygonal cells with irregular nuclei and large cytoplasm existed in the gaps between interdigitating reticulum cells (Fig. 5F, H). These polygonal cells were similar to cells observed in the spleens and MLNs of *Rag-1*-deficient pigs and exhibited morphologic characteristics reminiscent of immature lymphocytes.

### Immunohistochemical analysis of *Rag* ^−/−^ neonatal piglets

Furthermore, we confirmed lymphocyte development in the thymus, spleen, and MLNs by immunohistochemistry using CD3 as a lymphocyte marker (Fig. S1). There were numerous CD3^+^ T cells in the medulla of the thymus (Fig. S1A), the periarterial lymphatic sheaths of the spleen (Fig S1C), and the surrounding lymphoid follicles of MLNs (Fig. S1E) in wild-type piglets. In contrast to wild-type piglets, the thymus, spleen, and MLNs of the *Rag-1* KO piglets showed loss of CD3^+^ T cells (Fig. S1B, D and F).

## Discussion

In neonatal pigs, B cell development occurs earlier than T cell development and begins in the yolk sac [49], [50]. T cells are first detected in the thymus at a gestation of 40 days [49]. Peripheral blood IgM-positive B cells begin to appear at 40 days of gestation [49]. On the other hand, peripheral T cells are generated at 45 days of gestation [49]. In association with B lymphocyte development, the *Rag-1* gene is strongly expressed and V(D)J rearrangement starts at 20 days of gestation [50], and the rapid elevations of mRNA levels of *Rag-1, -2*, and *CD3ε* in the thymus increase from 40 to 65 days of gestation [51]. In this study, we generated *Rag-1*-deficient model pigs using gene targeting and SCNT and confirmed the contribution of the *Rag-1* gene to lymphogenesis and the phenotype of *Rag-1*-deficient pigs.

In mice, previous experiments have revealed that disruption of Rag-1 or Rag-2 blocks the differentiation of lymphocytes and causes T-B-SCID [6], [7]. Moreover, Rag-deficient blastocyst complementation allowed the generation of somatic chimeras possessing mature B and T cells, all of which were derived from injected ES cells [52]. In addition, studies on Rag activity in humans have revealed that Rag gene mutations cause T-B-SCID or Omenn syndrome [8]–[11]. Rag-1 and Rag-2 interact and the Rag complex functions as a sequence- and structure-specific nuclease during V(D)J recombination [53]. These reports suggest that Rag-1 and Rag-2 and their interaction are important for the differentiation and proliferation of T and B lymphocytes in rodents and humans. In this study, FACS analysis showed that, among PBMCs, IgM-positive B cells and CD3-positive T cells were present in wild-type neonatal piglets. In contrast, Rag-1 KO piglets completely lacked both IgM-positive B cells and CD3-positive T cells among their PBMCs. This suggests that the inactivation of Rag-1 blocks the differentiation and proliferation of T and B cells and Rag genes are important for the differentiation and proliferation of T and B lymphocytes in pigs, which is similar in mice and humans. These results confirm that Rag-1-deficient pigs exhibit the T-B-SCID phenotype.

In a recent study, Rag-1-deficient rats were reported by two groups [54], [55]. Although their Rag-1 mutant rats completely lacked Rag-1 protein and exhibited a hypoplastic thymus, they retained a small quantity of CD3-positive T cells or IgM-positive B cells [54], [55]. These differences in lymphocytes differentiation were ascribable to translation from an internal methionine without an N-terminal BIAA in the case of frame shift-mutated Rag-1 protein truncated in front of the nonamer binding region [56]. Our gene targeting scheme disrupted the coding sequence in Exon 2, 1.3 kb downstream from the initiator codon, leading to the inactivation of the porcine Rag-1 gene through antibiotic resistance genes similarly to Rag-1 KO mice [6]. Our results show that the function of Rag-1 gene was completely blocked on Rag-1 KO piglets.

Consistent with FACS analysis, histologic and immunohistochemistry analyses also showed a lack (abnormal development) of mature lymphocytes and structural defects in the spleen, MLNs, and thymus of the *Rag-1*-deficient piglets. *Immunoglobulin joining region gene (JH)* KO piglets devoid of B cells show a complete lack of follicular structure and germinal center organization in MLNs [57]. Moreover, *Il2rg-*disrupted pigs exhibit greatly reduced T cell development and hypoplastic white pulp in the spleen [38]. Furthermore, *Rag-1/-2* knockout pigs were recently reported to show a hypoplastic periarterial lymphatic sheath and loss of white pulp in the spleen as well as hypoplastic corpuscles in the thymus [41], [42]. Consistent with these lymphoid-defective pigs and previous reports on rodents, our *Rag-1* ^−/−^ neonatal piglets lacking T and B cells demonstrated hypoplasia of the white pulp and lymphoid follicles. Regarding the thymus, the architecture of the medulla and cortex and the lymphocytes were completely lost in *Rag-1* ^−/−^ piglets similar to the recent report [42]. Moreover, our *Rag-1* ^−/−^ neonatal piglets showed no CD3-positive cells in their thymus, spleen, or MLNs. These histologic findings also clearly prove that the characteristics of our *Rag-1* ^−/−^ neonatal piglets did not result from a leaky phenotype.

Unlike in rodents and *Rag-1 and -2*-biallelic mutant pigs, coat color changed in our *Rag-1* KO piglets. We used donor cells derived from Duroc fetuses. As shown in the wild-type figure (Figure 3A), Duroc piglets have a dark brown coat. On the other hand, the coat of *Rag-1*-deficient piglets was discolored. It is known that DNA methylation and epigenetic changes can be caused by SCNT [58]–[62], and phenotypic and epigenetic variation resulting in depigmentation has been reported in cloned pigs [63], [64]. In a study of piglets cloned using Duroc donor cells [64], one of nine cloned piglets exhibited depigmentation of the skin and hair. It is possible that the downregulation of certain genes induced this depigmentation, although the changes occurred during puberty. However, both our *Rag-1*-deficient piglets generated showed the same coat color accompanying the T-B-SCID phenotype. Therefore, the discoloration in *Rag-1*-deficient piglets may result from the *Rag-1* gene KO rather than an effect of cloning in colored pig breeds. The cause of this coat discoloration phenomenon is unknown, and further study is necessary to elucidate the relationship between coat coloration and *Rag-1* disruption in colored pigs.

Animals with SCID are a versatile model for laboratory, translational, and biomedical research. Furthermore, they may represent a platform for the development of humanized animals and the assessment of pluripotent stem cells. Although rodent models are widely used, human-scale models and long-term testing are still required to bridge the gap between animal models and humans. In this study, our findings provide additional evidence that the *Rag-1* gene is essential for lymphocyte development in pigs, which is similar in rodents and humans, and we confirmed that *Rag-1*-deficient pigs display a T-B-SCID phenotype with no leaks. Therefore, *Rag-1* KO pigs represent a promising model and provide important research resources that are similar to those described in recent reports. Future studies are required to confirm their utility as well as the fundamental characteristics of the phenotypic, immunologic, and morphologic changes in other breed and growth process.

## Supporting Information

Figure S1 Immunohistochemical analysis of *Recombination activating gene-1*-deficient piglets using antibodies recognizing CD3 (A and B) Immunohistochemical analysis of the thymus (×100). Abbreviations: CO, cortex; ME, medulla. (C and D) Immunohistolochemical analysis of the spleen (×400). Abbreviations: CA, central artery; PALS, periarterial lymphatic sheaths. (E and F) Immunohistochemistry of the mesenteric lymph nodes (×400). Abbreviations: LF, lymphoid follicle. These samples were immunostained with polyclonal rabbit anti-human CD3 (Dako, Glostrup, Denmark) diluted 1∶200 with PBS, and detected using the Envision+ system (Dako, Glostrup, Denmark). The ellipses identify CD3-positive cells.
(TIF)
