# Supplementary figures and images for: Generation of Recombination Activating Gene-1-Deficient Neonatal Piglets: A Model of T and B Cell Deficient Severe Combined Immune Deficiency
Source: PLoS One. 2014 Dec 1;9(12):e113833. doi: 10.1371/journal.pone.0113833 (PMC4249935; doi:10.1371/journal.pone.0113833)

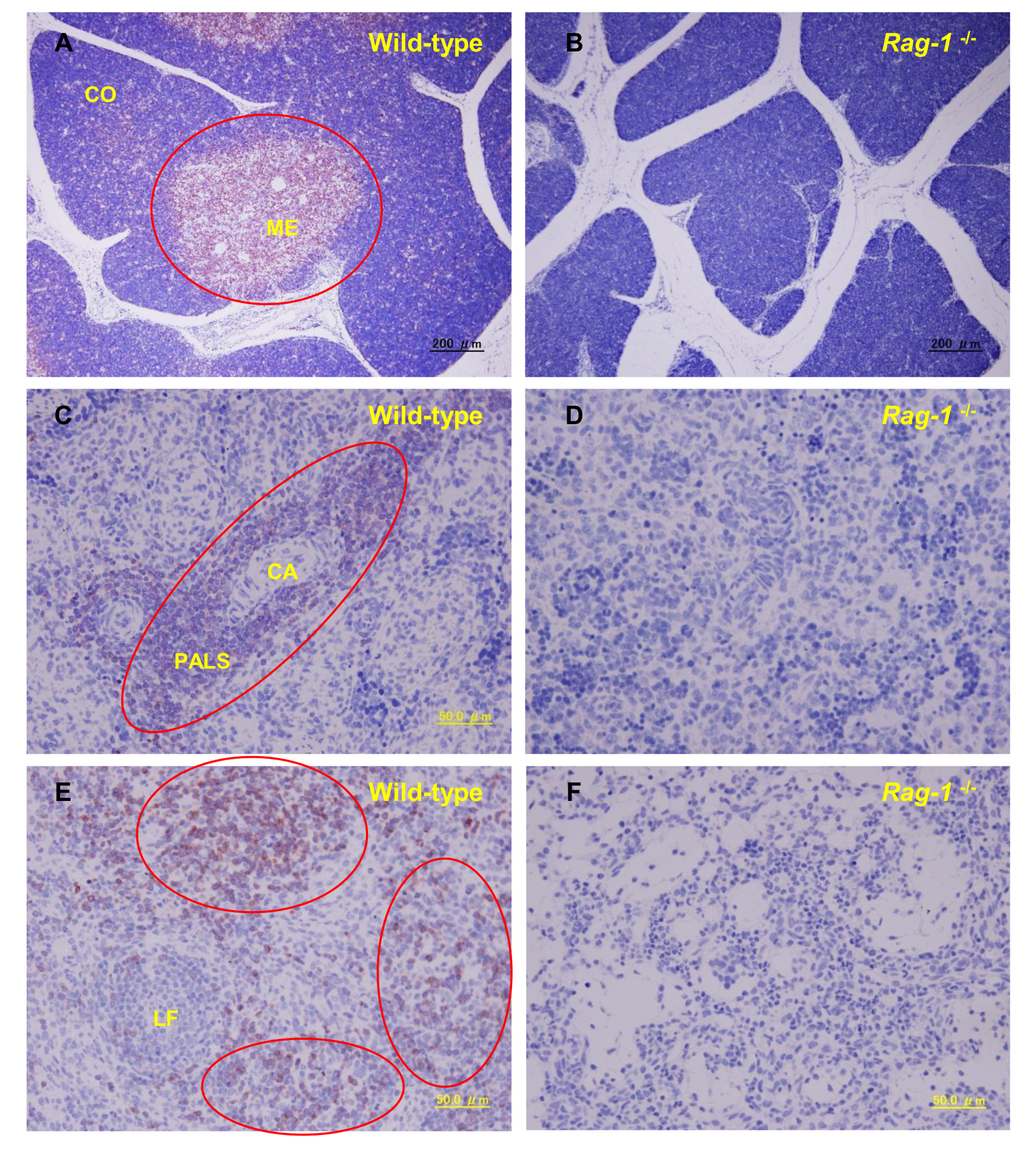

Supplement: Figure S1 — Immunohistochemical analysis of Recombination activating gene-1-deficient piglets using antibodies recognizing CD3 (A and B) Immunohistochemical analysis of the thymus (×100). Abbreviations: CO, cortex; ME, medulla. (C and D) Immunohistolochemical analysis of the spleen (×400). Abbreviations: CA, central artery; PALS, periarterial lymphatic sheaths. (E and F) Immunohistochemistry of the mesenteric lymph nodes (×400). Abbreviations: LF, lymphoid follicle. These samples were immunostained with polyclonal rabbit anti-human CD3 (Dako, Glostrup, Denmark) diluted 1∶200 with PBS, and detected using the Envision+ system (Dako, Glostrup, Denmark). The ellipses identify CD3-positive cells. (TIF) [file pone.0113833.s001.tif]
